# Dysmenorrhea Heat Therapy Injury in a Post-Abdominoplasty Patient: A Case Report

**DOI:** 10.7759/cureus.40169

**Published:** 2023-06-09

**Authors:** Hesham Alokaili, Maha Hanawi, Fatima Aldaker, Yara Alokaili, Zainab Alfaraj, Tanveer A Bhat, Anas Aljasir

**Affiliations:** 1 Department of Plastic and Reconstructive Surgery, King Saud Medical City, Riyadh, SAU; 2 College of Medicine, Alfaisal University, Riyadh, SAU; 3 College of Medicine, Dar Al Uloom University, Riyadh, SAU

**Keywords:** hypesthesia, home remedy, menstrual pain, burn injury, aesthetic abdominoplasty

## Abstract

The anterolateral abdominal wall has rich neurosensory innervation from many sensory nerves, and in abdominoplasty surgical procedures, these nerves are invariably cut, resulting in anesthesia or hypoesthesia in their respective territories. Here we report a 26-year-old healthy female post-abdominoplasty patient who sustained an incidental contact burn injury from a common home remedy for her menstrual pain. Fortunately, the burn healed with secondary intent. Post-surgical loss of protective sensation facilitated this injury from heat therapy for spasmodic dysmenorrhea. Therefore, the patients planned for abdominoplasty should be informed in advance about the possibility of the development of this complication with its associated sequelae and its prevention. Early recognition of this surgical complication and timely intervention will prevent the consequent disfiguring of the rejuvenated abdominal wall.

## Introduction

Among the well-known complications of abdominoplasty, nerve injury is little discussed in the available literature, besides being underestimated in terms of its morbidity. In the standard abdominoplasty technique, the surgeon makes the incision along the inferior aspect of the anterolateral abdominal wall, thereby putting nerves at risk of injury. Injury to these nerves may result in chronic pain, hypoesthesia, and paralysis and can severely affect a patient's quality of life (QoL) [1-3].

Hypesthesia is a common complication after abdominoplasty. It’s a result of denervation during the elevation of the cutaneous abdominal flap from underlying muscles. Ahrerra et al. reported that although this complication is well tolerated, with most patients being indifferent towards hypesthesia, it still predisposes them to incidental trauma [4]. Therefore, counseling patients about hypesthesia is recommended to avoid harmful, deforming traumas as well as for medico-legal purposes.

Hypesthesia is not only a potential risk factor for incidental trauma, but it may also jeopardize the post-operative cosmetic results because of the eventual post-traumatic scar formation and disfigurement. Thermal injury, in particular, may lead to disfiguring dyspigmentation and scarring. Loss of protective sensation makes previously harmless activities such as cooking and ironing potentially injurious. Similarly, we describe in this paper a burn from the often innocuous practice of heat therapy for menstrual pain in a post-abdominoplasty patient.

## Case presentation

**Figure 1:**
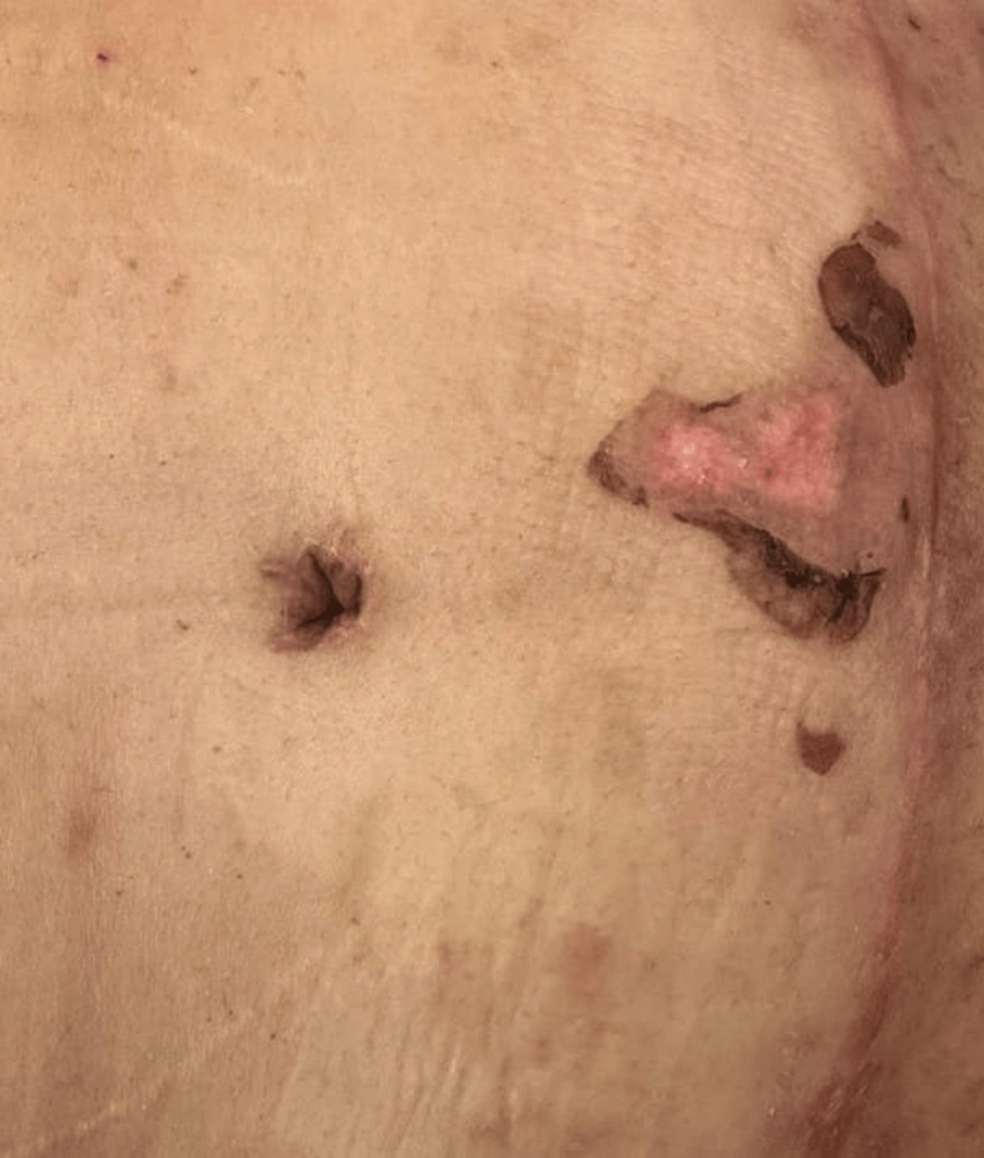
Superficial partial-thickness burn in the infra-umbilical region

A 26-year-old female asthmatic in remission with no prior surgical history presented to the clinic for treatment of abdominal redundancy after massive weight loss through lifestyle modification. A standard abdominoplasty was recommended and performed. The intraoperative and postoperative course was unremarkable, with one exception being incidental thermal trauma made possible by post-surgical abdominal hypesthesia (Figure 1).

**Figure 2:**
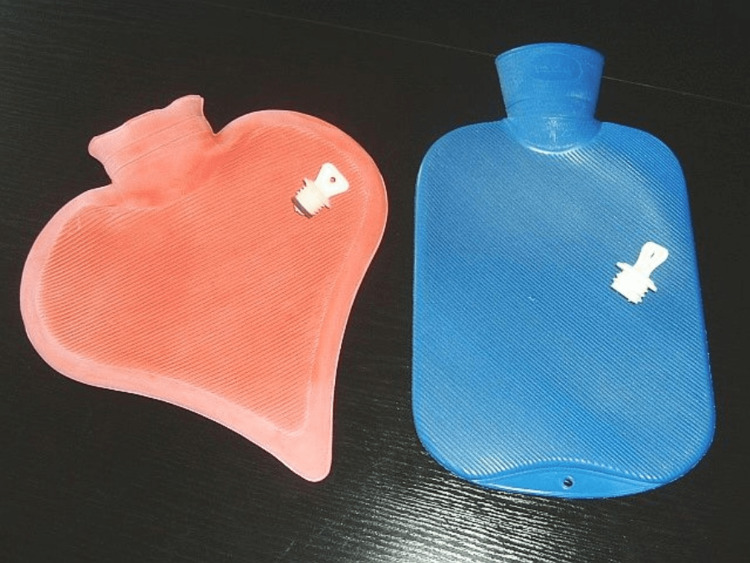
Hot water bottles are used in heat therapy for dysmenorrhea

At the clinic two weeks post-operatively, the patient was seen in good health and compliant with her compression garment. Locally, the surgical area was healing well, with no collections or dehiscence, but a thermal injury was discovered over the infra-umbilical area. From the history obtained, she sustained a superficial partial-thickness contact burn from a hot external bottle used in relieving menstrual pain (Figure 2). The injury was treated in standard fashion with irrigation, petroleum gauze dressings, and topical antimicrobials.

## Discussion

Abdominoplasties and other body contouring procedures are popular and have a positive impact on quality-of-life measures in the psychological, social, occupational, and sexual domains [5-6]. Cutaneous hypesthesia is a common complication of abdominoplasty under study [4,7,8]. Cutaneous innervation of the abdominal wall arises from the subcostal, iliohypogastric, ilioinguinal, and the lower five intercostal nerves [1,6]. These nerves are divided into lateral and anterior branches that perforate at the ribcage’s midaxillary line and through the rectus sheath, respectively [9].

Multiple studies in the literature have evaluated this complication using objective and subjective means. [4,7,8]. Hypoesthesia post-abdominoplasty may occur in all regions, but it’s most pronounced in the midline infraincisional, infraumbilical, and supraumbilical zones [4,8]. Supplying these regions are the anterior branches, comprised of the lower intercostal nerves, and their division is inherent to the procedure described by Duchateau et al. [10]. While the upper and lateral branches are relatively more spared due to comparatively limited dissection and their subcutaneous course.

In particular, the most commonly and severely affected region is the infraumbilical zone [4,7]. Farah et al. used all sensory modalities to evaluate hypoesthesia [7]. The infraumbilical zone had the most profound loss of sensation to pressure [7]. Furthermore, Farah et al. reported decreases in all other sensory modalities within this zone, including temperature, pain, and superficial touch [7]. These sensory losses undoubtedly predispose to incidental trauma from lost protective sensation. Especially in the context of strict compression garment compliance and wound dressings, which may keep these injuries hidden and unnoticed.

Such as in our patient who sustained a superficial partial thickness contact burn that had gone unnoticed during heat therapy for dysmenorrhea. Potur et al. reported heat therapy to be a common relieving practice used by as many as 36%-50% of women [11]. It is believed to relieve pain through attenuation of muscle tension and facilitating outflow of pelvic tissues, which may reduce edema and associated nerve compression [12,13]. Given the popularity of this remedy and the vulnerability of this zone to incidental trauma, we recommend counseling about caution or avoidance of this practice in post-abdominoplasty patients.

## Conclusions

Post-surgical hypoesthesia predisposes to incidental trauma, and it is most profound in the infraumbilical zone after abdominoplasty. Heat therapy, a popular treatment of dysmenorrhea, targets this vulnerable zone and is thus potentially harmful without protective sensation. Therefore, we recommend counseling for caution or avoidance of this practice in post-abdominoplasty patients. Especially given the possibly disfiguring complications of thermal injury.
